# The Effects of Marine Algal Polyphenols, Phlorotannins, on Skeletal Muscle Growth in C2C12 Muscle Cells via Smad and IGF-1 Signaling Pathways

**DOI:** 10.3390/md19050266

**Published:** 2021-05-10

**Authors:** Seo-Young Kim, Ji-Hyeok Lee, Nalae Kang, Kil-Nam Kim, You-Jin Jeon

**Affiliations:** 1 Department of Marine Life Science, Jeju National University, Jeju 63243, Korea; kimmsy1127@naver.com or; 2 Chuncheon Center, Korea Basic Science Institute (KBSI), Chuncheon 24341, Korea; knkim@kbsi.re.kr; 3 Natural Products Research Division, Honam National Institute of Biological Resources (HNIBR), 99, Gohadoan-gil, Mokpo 58762, Korea; lee198186@hanmail.net; 4 Jeju Research Institute, Korea Institute of Ocean Science and Technology, Jeju 63349, Korea; nalae1207@kiost.ac.kr

**Keywords:** muscle growth, myogenesis, marine algae, *Ecklnoia cava*, marine algal polyphenols, phlorotannins

## Abstract

Skeletal muscle is an important tissue in energy metabolism and athletic performance. The use of effective synthetic supplements and drugs to promote muscle growth is limited by various side effects. Moreover, their use is prohibited by anti-doping agencies; hence, natural alternatives are needed. Therefore, we evaluated the muscle growth effect of substances that can act like synthetic supplements from edible marine algae. First, we isolated six marine algal polyphenols belonging to the phlorotannin class, namely dieckol (DK), 2,7″-phloroglucinol-6,6′-bieckol (PHB), phlorofucofuroeckol A (PFFA), 6,6′-bieckol (6,6-BK), pyrogallol-phloroglucinol-6,6′-bieckol (PPB), and phloroglucinol (PG) from an edible brown alga, *Ecklonia cava* and evaluated their effects on C2C12 myoblasts proliferation and differentiation. Of the six phlorotannin isolates evaluated, DK and PHB induced the highest degree of C2C12 myoblast proliferation. In addition, DK and PHB regulates myogenesis by down-regulating the Smad signaling, a negative regulator, and up-regulating the insulin-like growth factor-1 (IGF-1) signaling, a positive regulator. Interestingly, DK and PHB bind strongly to myostatin, which is an inhibitor of myoblast proliferation, while also binding to IGF-1 receptors. Moreover, they bind to IGF-1 receptor. These results suggest that DK and PHB are potential natural muscle building supplements and could be a safer alternative to synthetic drugs.

## 1. Introduction

Muscle growth is regulated via a complex interaction of diverse biochemical signaling pathways during cell development [1,2]. In particular, myostatin, a member of the transforming growth factor-beta (TGF-β) family, is known to play a part in determining muscle growth by regulating muscle mass [3,4]. Myostatin bound to activin receptor type IIB (ActRIIB) triggers a signaling cascade, which can lead to inhibition of myoblast differentiation and proliferation [5]. However, specific inhibitors such as follistatin (Fst), follistatin-like 3 (Fstl3), Fst-type molecules including Fst288, and Fst315, and growth and differentiation factor-associated with serum protein 1 (GASP-1) can prevent the binding of myostatin to activin receptor type II B (ActRIIB) and inhibit the signaling cascade [3,6,7,8]. One of the main positive regulators of muscle growth is insulin-like growth factor 1 (IGF-1), which induces an increase in muscle mass by stimulating the Akt pathways [9]. The binding of IGF-1 to its receptors such as IGF-1 receptor triggers the activation of PI3K, which induces phosphorylation of Akt and leads to regulation and activity of the Forkhead box O (FoxO) family by phosphorylating them. During the proliferation of myoblast, FoxO1 and FoxO3 are localized in the cytoplasm in a latent phosphorylated form [10].

Phenolic compounds are secondary metabolites reported to have antioxidant, antimutagenic, anti-obesity, and anticarcinogenic effects and have been predicted to reduce the risk of disease and bring health benefits after daily intake [11]. Among the phenolic compounds, carnosic acid, isolated from rosemary (*Rosmarinus officinalis*), has been studied to induce muscle growth and differentiation in in vivo studies [12].

**Figure 1:**
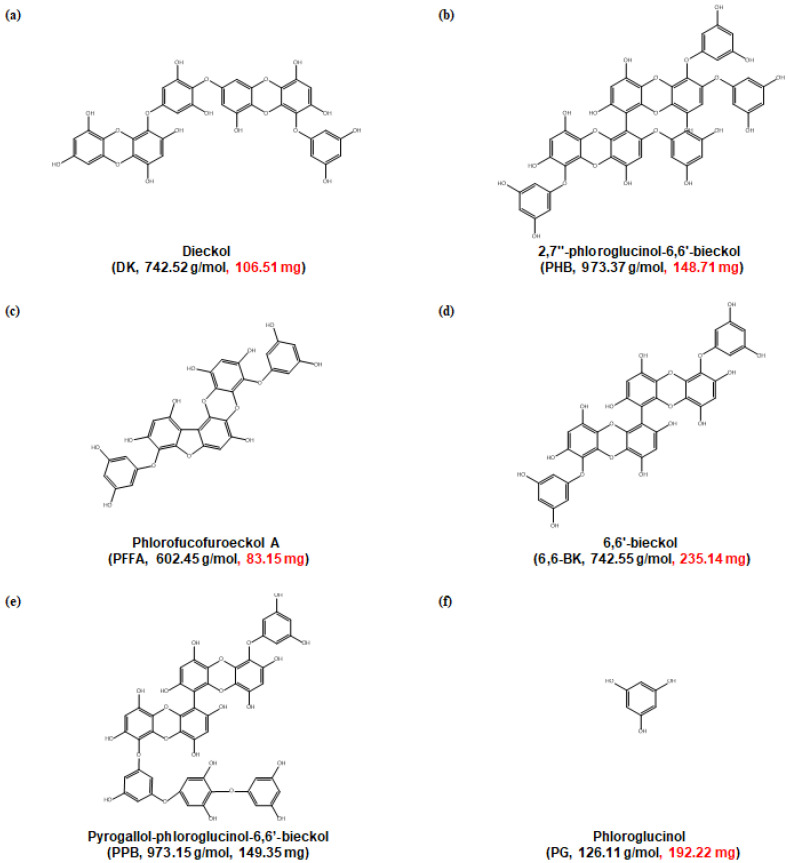
Chemical structures of phlorotannins from *E. cava* extract. The molecular weight and content of each phlrotannins are indicated in parentheses.

Brown marine algae have long been used as food-stuffs and folk medicines in Asian countries, such as Korea and Japan [13]. *Ecklonia* species and brown algae have been used extensively as a traditional medicine in countries for the treatment of goiter, scrofula, urinary diseases, stomach ailments, hemorrhoids, boils, laxative, and tonic for lying-in woman [14]. In particular, *Ecklonia cava* (*E. cava*) is widely distributed at the southern coasts of Korea and Japan, and utilized to produce food ingredients, animal feed, fertilizers and folk medicine in gynecopathy and so on [15]. *E. cava* contains two potential medicinal ingredients: polysaccharide and unique polyphenol, called “phlorotannins” [16]. In particular, *E. cava* is a rich phlorotannins, and includes eckol, DK, 6,6-BK, and PHB, which are formed by the polymerization of phloroglucinol monomer units [17]. Here, we isolated six phlorotannins containing DK, 6,6-BK, and PHB from *E. cava* (Figure 1). Additionally, many reports indicate that many of the medicinal properties of these species are derived from these polyphenols [18,19]. However, studies on its effect on skeletal muscle growth is scarce. In this study, therefore, we aimed to identify *E. cava*-derived phlorotannin substances that have effects similar to myostatin inhibitors and IGF-1 agonists.

## 2. Results

### 2.1. Cytotoxicity of Phlorotannins

**Figure 2:**
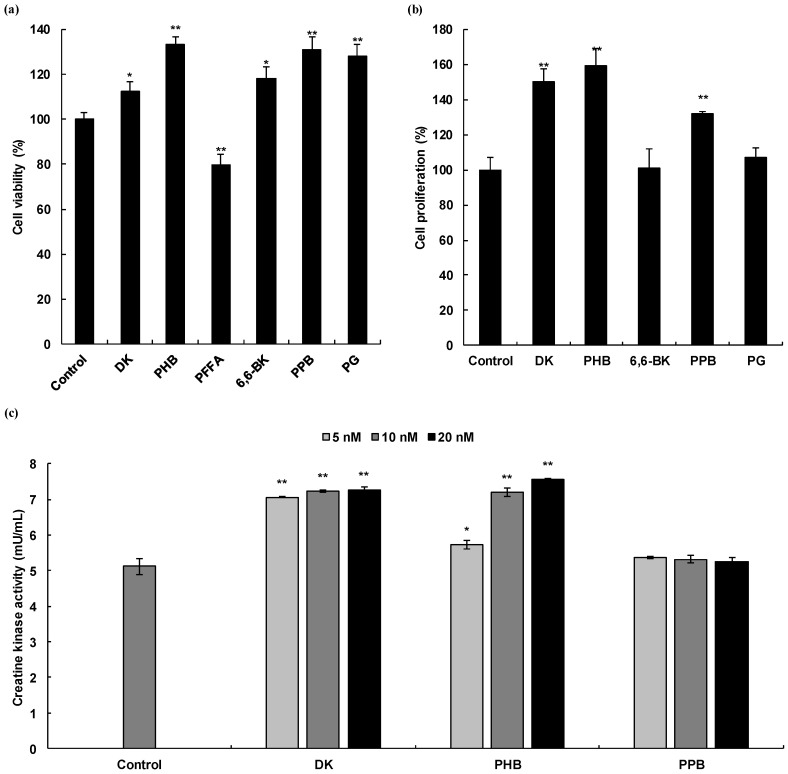
Skeletal muscle cell proliferation activities of phlorotannins isolated from *E. cava*. Cell viability of six phlorotannins and DHT on C2C12 myoblasts (**a**), Cell proliferation activity of five phlorotannins, which exhibit non-cytotoxicity on C2C12 myocytes (**b**), and Creatine kinase activity of DK, PHB, and PPB, which has cell proliferative effects (**c**). Experiments were performed in triplicate and the data were expressed as mean ± S.E.M.; * *p* < 0.05, and ** *p* < 0.01 as compared to the non-treated cells. DK, Dieckol; PHB, 2,7″-phloroglucinol-6,6′-bieckol; PFFA; Phlorofucofuroeckol A; 6,6-BK; 6,6′-bieckol; PPB; Pyrogallol-phloroglucinol-6,6′-bieckol; PG, Phloroglucinol.

Prior to evaluating the cell proliferation effects of phlorotannins obtained from *E. cava*, we first examined the cytotoxicity of the phlorotannins against C2C12 myoblasts using the MTT assay. Among the six phlorotannins investigated, all, with the exception of PFFA, induced cell proliferation rather than cell toxicity compared to untreated cells (Figure 2a).

### 2.2. Cell Proliferation Activity of Phlorotannins

To evaluate the cell proliferation activities of phlorotannins in C2C12 myocytes, we investigated the cell proliferative effects of phlortannins on C2C12 during the differentiation phase. As shown in Figure 2b, DK, PHB, and PPB significantly increased the proliferation of myocytes, as compared to that of the untreated cells and showed the same significance as the positive control, DHT.

### 2.3. Creatine Kinase (CK) Activity

CK activity, a well-described biomarker of C2C12 cell differentiation [20], was measured in the cell lysates treated with DK and PHB. CK is an enzyme (EC 2.7.3.2) expressed by various tissues and cell types. CK can catalyze the conversion of creatine and consumes adenosine triphosphate (ATP) to generate phosphocreatine (PCr) and adenosine diphosphate (ADP). This CK enzymes reaction is reversible and thus ATP can be generated from PCr and ADP. In particular, CK is an important enzyme in muscle tissue. Therefore, we assessed the CK activity of C2C12 myocytes during differentiation by DK, PHB, and PPB. As shown in Figure 2c, DK and PHB significantly increased CK activity in the cells compared to that of the control. In the case of PPB treated cells, a clear induction of CK activity was not observed.

### 2.4. Effects of Muscle Growth Regulation of DK and/or PHB

**Figure 3:**
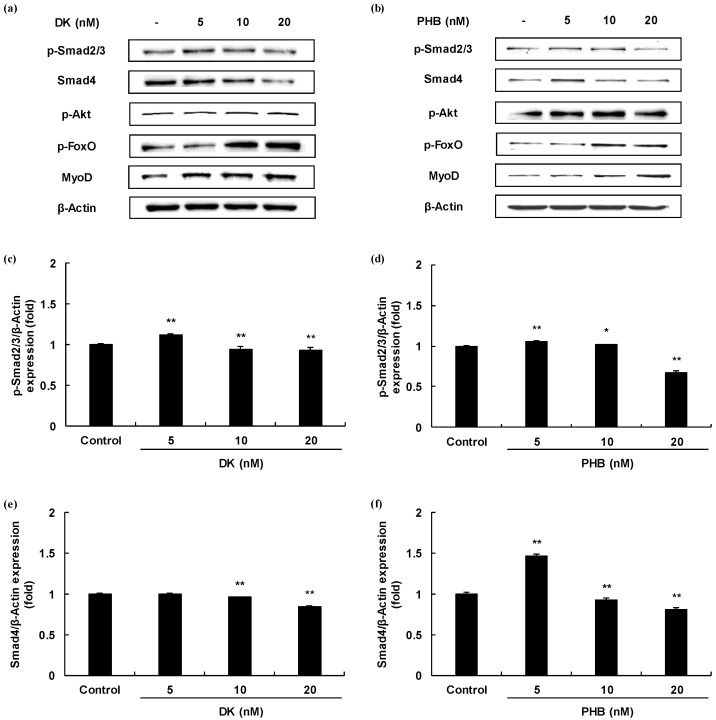

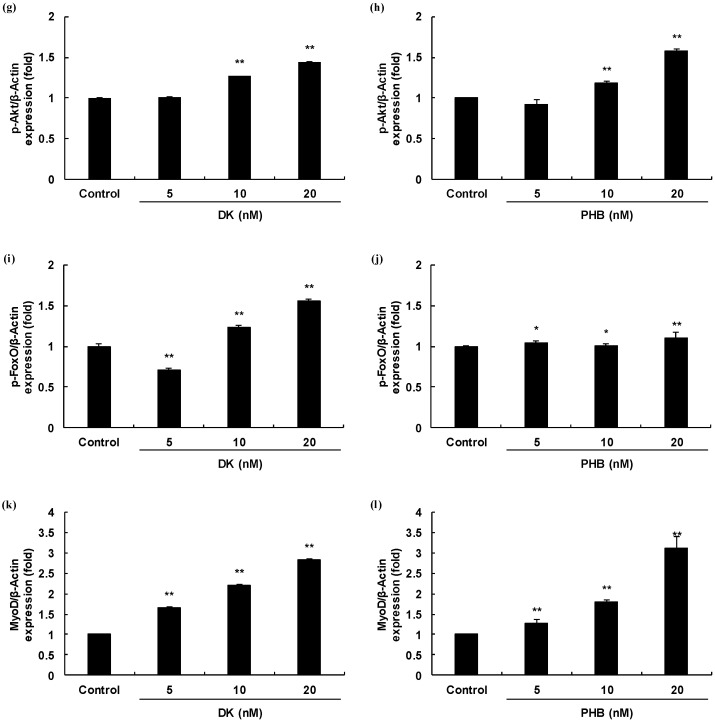
Skeletal muscle cells growth activity of DK and PHB on C2C12 myotubes. (**a**,**b**), Bands of protein expressions; (**c**–**f**), TGF-β such as myostatin mediated Smad proteins expressions (p-Smad2/3, and Smad4); (**g**–**j**), IGF-1 mediated proteins expressions (p-Akt, and p-FoxO); (**k***,***l**), MyoD protein, which is known to play a critical function in the regulation of muscle cell development expressions. Experiments were performed in triplicate and the data were expressed as mean ± S.E.M.; * *p* < 0.05, and ** *p* < 0.01 as compared to the control group.

Myogenesis differentiation and growth mediated intracellular signaling of the TGF-β superfamily and IGF-1. In order to determine, therefore, whether two active phlorotannins (DK, and PHB) can regulate the TGF-β and/or IGF-1 mediated signaling pathways, Western blot analysis was carried out and the results are shown in Figure 3. First, we analyzed the protein expressions level of Smad proteins regulated by TGF-β such as myostatin. The protein expressions levels of p-Smad2/3 of DK or PHB-treated cells were significantly decreased. And, the Smad4 protein expressions levels also were significantly reduced by both DK and PHB. In the case of PHB treatment, the lowest concentration of PHB (5 nM) had no effect on the decrease in the expression level of Smad4 protein, but rather increased the expression level compared to the control. However, treatment with other concentrations (10 and 20 nM) significantly reduced the protein expression level of Smad4 compared to control. Next, we assessed the p-Akt/p-FoxO proteins expressions level regulated by IGF-1 in the DK or PHB treated cells during differentiation period. As shown in Figure 3g–j, DK and PHB significantly increased p-Akt protein as well as p-FoxO protein. Furthermore, MyoD, which is known to play a critical function in the regulation of muscle cell development, significantly increased following treatment with DK or PHB (Figure 3k,l).

### 2.5. Anaylsis of In Silico Molecular Docking of Phlorotannins with TGF-β (Myostatin)

**Figure 4:**
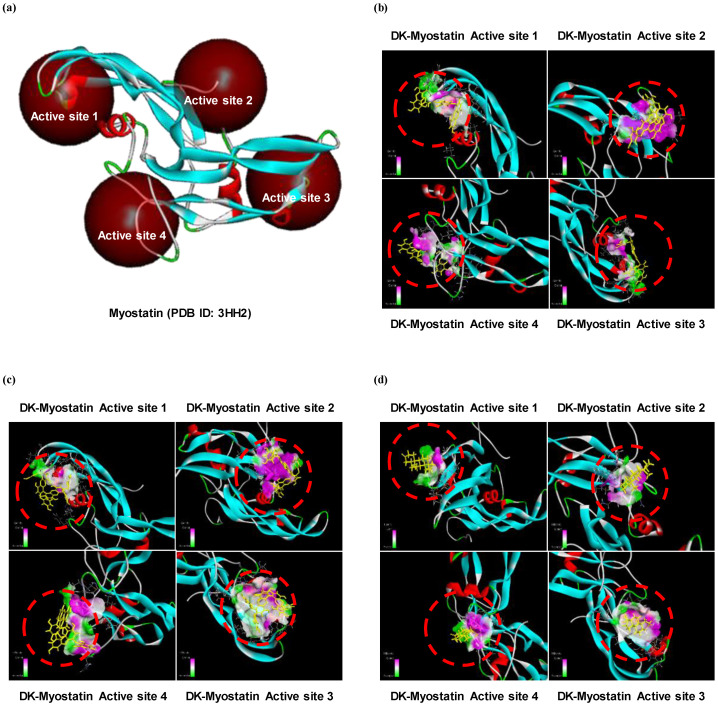
Computational prediction of binding 3 dimensional structures by docking simulations of two active phlrotannins (DK and PHB) and DHT with myostatin. (**a**) Crystal structure of myostatin allocated from protein data bank (PDB ID: 3HH2); (**b**) DK-myostatin complexes; (**c**) PHB-myostatin complexes; and (**d**) DHT-myostatin complexes. DHT is dihydrotestosterone as a positive control.

We have shown that the level of protein expressions of Smad proteins is reduced in DK or PHB-treated cells. Therefore, we tried to determine whether these two active phlorotannins could not only reduce the expression of Smad proteins, but also inhibit the activation of TGF-β, which activates them. Therefore, the simulation of docking with TGF-β superfamily, myostatin and active phlorotannins (DK or PHB) was performed. As a result, docking of the ligands-protein (phlorotannins-myostatin) complexes was successful as DK or PHB stably posed into the myostatin active sites (Figure 4). In addition, DHT, which was used a positive control, was docked with four active sites of myostatin. Moreover, it was shown that DK and PHB binds to myostatin at lower binding energy values than DHT (Table 1).

### 2.6. Anaylsis of In Silico Molecular Docking of Phlorotannins with IGF-1 Receptor (IGF-1R)

**Figure 5:**
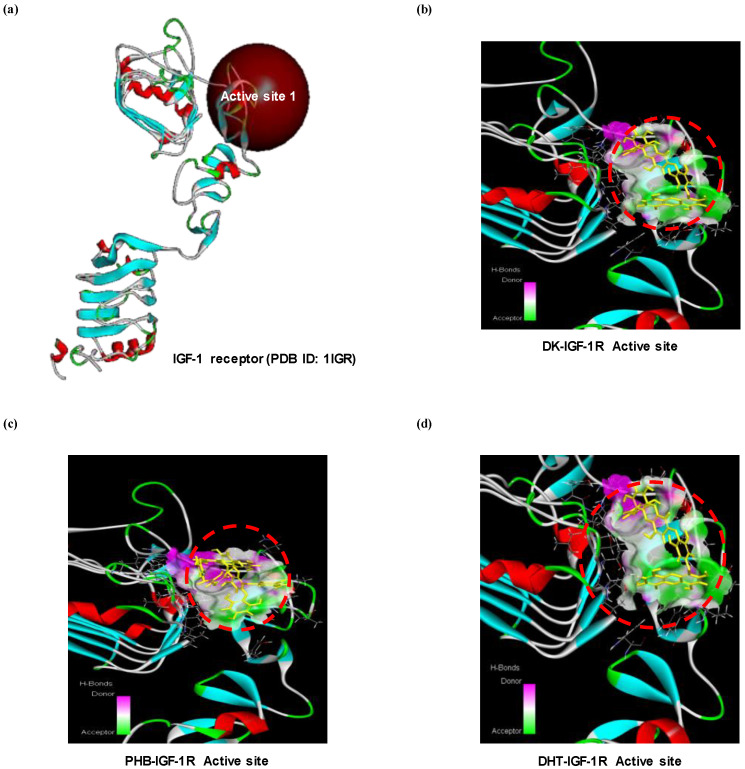
Computational prediction of binding 3 dimensional structures by docking simulations of two active phlrotannins (DK and PHB) and DHT with IGF-1R. (**a**) Crystal structure of IGF-1R allocated from protein data bank (PDB ID: 1IGR); (**b**) DK-IGF-1R complexes; (**c**) PHB-IGF-1R complexes; and (**d**) DHT-IGF-1R complexes. DHT is dihydrotestosterone as positive control.

As shown above, DK and PHB increased the level of proteins expressions regulated by IGF-1. Therefore, to explore a IGF-1 agonist from these two active phlorotannins, we performed a docking simulation with IGF-1 receptor and phlorotannins. As shown in the Figure 5, docking of the ligands-protein (phlorotan-nins-IGF-1R) complexes was successful as DK or PHB stably posed into the IGF-1R active site (cys-rich sites). Additionally, as shown in Table 2, DHT was docked into IGF-1R with a higher binding energy value (−60.25 kcal/mol) than DK (−118.71 kcal/mol) and PHB (−98.11 kcal/mol).

## 3. Discussion

Skeletal muscle is the dominant organ system in locomotion and energy metabolism [21]. In humans, muscle development is regulated by maintaining a delicate balance between hyperplasia and hypertrophy via a network of diverse physiological signaling pathways. Myostatin, one of the TGF-β superfamilies, is a potent negative regulator of skeletal muscle growth and maintenance [5,20]. Mutation of the myostatin gene has been reported to cause a dramatic increase in muscle mass in mice, cattle, sheep, and humans [22]. Active myostatin mostly binds to ActRIIB receptors and triggers a signaling cascade, resulting in phosphorylation of the two serine residues of Smad2 or Smad3 at the COOH domains and assembly of Smad2/3 with Smad4. This signaling pathway leads to active transcription of target genes [5]. On the other hand, when IGF-1 binds to IGF-1R, it induces phosphorylation of Akt and FoxO. This signaling cascade eventually increases muscle development [2,23,24]. In the present study, we aimed to identify the inducing muscle growth effect from natural bioactive phlorotannins, which are contained only in marine algae, to acts as myostatin inhibitors or IGF-1 agonists. Therefore, we carried out myostatin mediated Smad proteins expressions and IGF-1 mediated phosphorylated Akt and FoxO proteins expressions through Western blot analysis. As a result, DK reduced the protein expressions level of p-Smad2/3 and Smad4 except the lowest concentration (5 nM). In the case of PHB treatment, a high concentration of PHB (20 nM) significantly reduced the p-Smad2/3 and Smad4 expressions level rather than DK. These results show that PHB has more influence on the reduction of the expressions of Smad proteins than DK. The protein expressions levels of p-Akt and p-FoxO were also significantly increased by both DK and PHB treatment. However, the level of p-Akt expression was increased in the PHB treated cells rather than DK, and that of p-FoxO was increased by DK than PHB. Taken together, both DK and PHB regulate the expression levels of Smad proteins and p-Akt/p-FoxO proteins, but in the case of Smad proteins, it is believed that it is more regulated by PHB than by DK. Additionally, we assessed the expression level of MyoD, a protein in animals that plays a major role in regulating muscle development and takes part in the repair of damaged skeletal muscle [25,26,27]. The levels of MyoD protein expression were both concentration-dependently increased by DK or PHB treatment.

We moved to explore further in order to determine how these phlorotannins interact with myostatin and/or IGF-1R, which are involved in the expression of above proteins. To do this, we first analyzed how myostatin and IGF-1R are structured in myocytes. Next, molecular docking simulation was performed. Myostatin has a traditional TGF-β family hand-shaped structure consisting of four curved beta strands (fingers), a cysteine knot motif in the ‘palm’ region, and a major helix (wrist) [8]. However, it is Fst-type molecules, such as Fst288, Fst35, or Fstl3, that act as antagonists of this myostatin, showing that they completely surround the ligand and block all four receptor binding sites, thereby inhibiting signal transduction [8]. Therefore, we analyzed whether DK or PHB binds to the four sites of myostatin like the Fst-type molecules. As a result, both DK and PHB stably bound to the four sites (here, each active site is designated as active site 1, 2, 3, and 4) of myostatin. IGF-1R consists of three domains, L1-Cys-rich-L2. The L domains each consist of single-stranded right-handed β-helix [28]. The Cys-rich region is composed of eight disulphide-bonded modules, seven of which form a rod-shaped domain with associated modules in an unusual manner [28]. The complexes of the ligands and IGF-1, IGF-2, and insulin share a common architecture and competitively cross-react with IGF-1R and IR [28]. Primarily, the specificity determinants for IGF-1 binding are known to be located in the Cys-rich domain [25]. Based on the above information, the simulation of ligands-IGF-1R complexes was performed by binding to the Cys-rich region. As a result, DK and PHB also stably combined with IGF-1R.

Overall, DK and PHB, phlorotannins isolated from *E. cava* act as myosatin inhibitors and IGF-1 agonist, and eventually have a positive effect on muscle growth.

## 4. Materials and Methods

### 4.1. Preparation of Crude Extract and Phlorotannins from E. cava

The marine brown alga *E. cava* was collected along the coast of Jeju Island, Korea. The dried *E.* cava powder (500 g) was extracted with 70% (*v*/*v*) aqueous ethanol (5 L) and under stirring at room temperature (RT) for 24 h. After that, the extract was filtered and the filtrate was evaporated under reduced pressure at 40 °C in order to obtain an ethanol extract, which was suspended in water, then extracted with ethyl acetate (EtOAc). The EtOAc extract (46.27 g) was subjected to Sephadex LH-20 column, and then finally purified by reversed-phase high performance liquid chromatography (HPLC) and quadrupole mass detection (QDa detector, Waters, MA, USA) to obtain compounds (Figure S1). HPLC analysis was performed on a Waters HPLC system, mobile phase consisted of methanol (MeOH)-water with a gradient method (0 min 20:80 *v*/*v*; 0–25 min 20:80–40:60 *v*/*v*; 25–27 min 40:60–20:80 *v*/*v*; 27–35 min 20:80–20:80 *v*/*v*). The column was poroshell EC C18 (4 μm, 4.6 × 100 mm column, Agilent Technologies, Santa Clara, CA, USA), and flow rate was 0.5 mL/min. The results were checked at 230 nm range. Mass spectral data were analyzed on electro spray ionization (ESI) interface mode and the operating conditions were negative ionization mode, spray voltage 5 kV, capillary temperature 275 °C, collision energy 35 eV. Mass spectra data were analyzed in the mass range of *m*/*z* 100–1200 (Figure S2), and compared with the data in the literature [29,30,31,32]. Eventually, it was determined as corresponding compounds, DK (106.51 mg), PHB (148.71 mg), PFFA (83.15 mg), 6,6-BK (235.14 mg), PPB (149.35 mg), and PG (192.22 mg) (Figure 1).

### 4.2. Skeletal Muscle Cell Grwoth Activities of Phlorotannins

#### 4.2.1. Myoblasts Cell Culture and Differentiation to Myocytes

The myoblasts used in this study were mouse-derived C2C12 cells, and the method for cell culture and differentiation followed the method described by Kim et al. [33].

#### 4.2.2. Cytotoxicty of Phlorotannins

The cytotoxic assessment was performed using MTT assay. Briefly, after 24 h of cell seeding (1.0 × 10^5^ cells/mL into 96 well plate), isolated phlorotannins (10 nM) were treated for an additional 24 h. After, MTT stock solution (50 μL, 2 mg/mL in DPBS) was added to each well for 4 h 37 °C. Then, the absorbance of the formazan crystals dissolved by DMSO at 540 nm was measured using the ELISA plate reader (BioTek Instruments, Inc., Winooski, VT, USA). Additionally, the survival rate of phlorotnanins-treated cells was calculated and compared to 100% of non-treated cells.

#### 4.2.3. Cell Proliferation Activity of Phlorotannins

To assess the skeletal muscle cell proliferation effects of the phlorotannins on C2C12 cells, acti were determined by the MTT assay using the procedure described by Ko et al. [34]. During the differentiation period, once every two days, the medium was replaced with a differentiation medium (DMEM containing 2% HS, and 1% streptomycin-penicillin) and phlorotnanins were treated. After that, cell proliferation was measured at the 540 nm absorbance; cell proliferation activity of phlorotannins-treated cells was calculated and compared to 100% of non-treated cells.

#### 4.2.4. CK Activity of Phlorotannins

To determine CK activity of phlorotannins, cell lysates treated with phloroatnnins during differentiation period were centrifuged at 12,000 rpm for 5 min, and the supernatants were assessed using the NADPH-coupled assay (BioVision, Inc., Milpitas, CA, USA). The NADPH generated by CK reaction was measured by determining the absorbance at 450 nm using and compared to non-treated control cells.

### 4.3. Effects of Phlorotanins on Skeletal Muscel Cells Differentiation

#### 4.3.1. Western Blot Analysis

Western blot analysis was carried out according to the protocol described by Kim et al. [33] in order to determine whether potential active phlorotannins affect the expression levels of proteins involved in muscle differentiation. The membranes containing these proteins were exposed to FUSION Solo6S program equipped with eVo-6 camera (Vilber Lourmat, Marne-La-Vallée, Collégien, France). Additionally, the basal levels of each protein were normalized by analyzing the level of the β-Actin protein by using the Image J program.

#### 4.3.2. In Silico Molecular Docking Simulation

To verify the potential active phlorotannins that can act as myostatin inhibitors and/or IGF-1R agonists, a molecular docking study was performed. First, the crystal structure of myostatin (PDB ID: 3HH2) and IGF-1R (PDB ID: 1IGR) were allocated from Protein Data Bank (PDB, http://www.pdb.org (accessed on 1 May 2019)). Then, a docking simulation was performed using a flexible docking tool in Accelrys Discovery Studio (DS) 3.5 (BIOVIA, San Diego, CA, USA) to see if the potential active phloroatnnins bind to myostatin and/or IGF-1R. Additionally, the values of the binding energies for their binding were calculated in DS 3.5.

### 4.4. Statistical Analysis

All the experiments were performed in triplicate and presented data as mean ± standard error (SE). Statistical analysis was conducted using a one-way analysis of variance (ANOVA) test (using SPSS 12.0 statistical software). Significance among the treatments was determined by using the Tukey test to analyze the difference. A *p*-values of less than 0.05 (*p* < 0.05) and 0.01 (*p* < 0.01) was considered to be significant.

## 5. Conclusions

In this study, we investigated the muscle growth activity of the phlorotannins isolated from *E. cava*. Especially, DK and PHB exhibited cell proliferation effects and CK activity without cytotoxicity. These two active phlorotannins decreased the expressions level of Smad proteins, which are mediated by TGF-β, such as myostatin, to activate the suppression of muscle differentiation and synthesis. In addition, the expressions level of p-Akt/p-FoxO proteins due to the binding of IGF-1 and IGF-1R to activate muscle synthesis were increased by DK and PHB. Furthermore, DK and PHB docked into myostatin and IGF-1R, and this result supports the change in the expression level of intracellular proteins. Taken together, we suggest that DK and PHB from marine alga *E. cava*, might be useful as potential muscle growth alternatives of synthetic steroid hormones with the Fst family acting as a myostatin inhibitor or agonist acting as IGF-1. However, these phlorotannins, which have already been proven to be beneficial to health through various biological activities, are non-toxic to muscle cells and induce muscle cell growth, but additional in vivo tests are required.

## Figures and Tables

**Table 1:** Results of docking simulations of two active phlorotannins (DK and PHB) with myostatin (PDB ID: 3HH2). DHT is dihydrotestosterone as a positive control.

Receptor (Binding Site)	Ligand	Binding Energy (kcal/mol)	-CDOCK Interaction Energy (kcal/mol)
Myostatin (3HH2-Active site 1)	DK	−146.06	50.77
	PHB	−109.90	42.44
	DHT	−50.00	24.07
Myostatin (3HH2-Active site 2)	DK	−118.99	40.01
	PHB	−102.71	49.42
	DHT	−48.16	18.28
Myostatin (3HH2-Active site 2)	DK	−126.49	47.71
	PHB	−143.74	57.70
	DHT	−51.51	32.07
Myostatin (3HH2-Active site 2)	DK	−108.64	38.27
	PHB	−125.90	54.60
	DHT	−40.08	18.76

**Table 2:** Results of docking simulations of two active phlorotannins (DK and PHB) with IGF-1R (PDB ID: 1IGR). DHT is dihydrotestosterone as positive control.

Receptor (Binding Site)	Ligand	Binding Energy (kcal/mol)	-CDOCK Interaction Energy (kcal/mol)
IGF-1R (1IGR-Active site)	DK	−118.71	50.66
	PHB	−98.11	60.74
	DHT	−60.25	20.33

## Supplementary Materials

The following are available online at https://www.mdpi.com/article/10.3390/md19050266/s1, Figure S1: The high-performance liquid chromatography (HPLC) chromatogram of ethyl acetate (EtOAc) fraction from 70% (*v*/*v*) aqueous ethanol extract of *E. cava*, Figure S2: The mass spectral data were analyzed on electro spray ionization (ESI) interface mode of six peaks obtained from LC/MS.
