# Supplementary material for: The Effects of Marine Algal Polyphenols, Phlorotannins, on Skeletal Muscle Growth in C2C12 Muscle Cells via Smad and IGF-1 Signaling Pathways
Source: Mar Drugs. 2021 May 10;19(5):266. doi: 10.3390/md19050266 (PMC8150305; doi:10.3390/md19050266)

Figure S1. The high-performance liquid chromatography (HPLC) chromatogram of ethyl acetate (EtOAc) fraction from 70% (*v*/*v*) aqueous ethanol extract of *E. cava*.

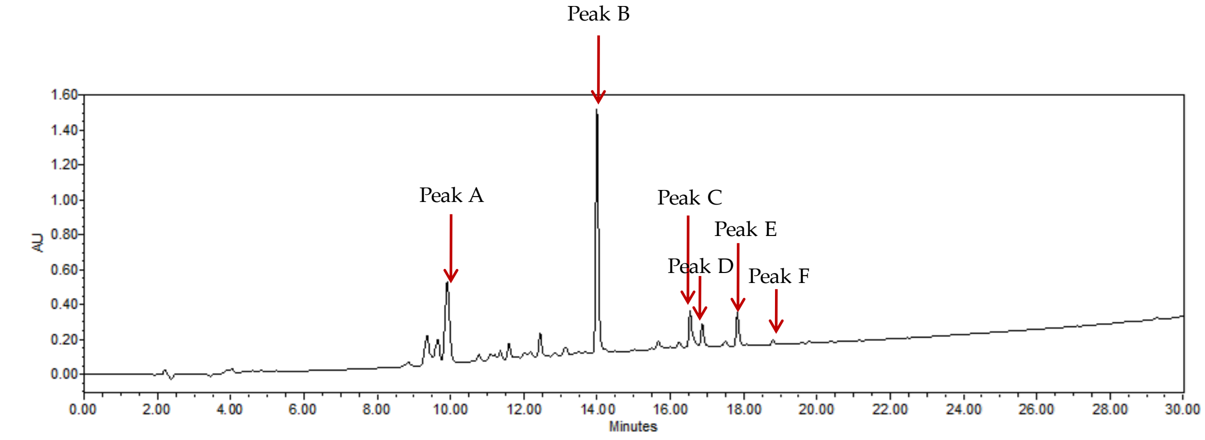


Figure S2. The mass spectral data were analyzed on electro spray ionization (ESI) interface mode of six peaks obtained from LC/MS.

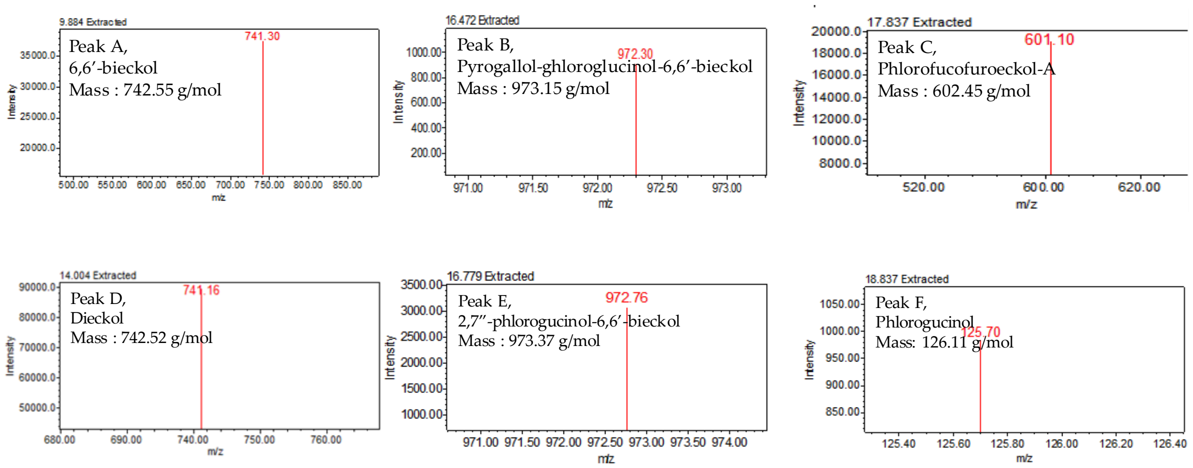

Supplement: Supplementary file 1 [file marinedrugs-19-00266-s001.zip › marinedrugs-1203809-supplementary.pdf]
